# The lncRNA GATA3-AS1/miR-495-3p/CENPU axis predicts poor prognosis of breast cancer via the PLK1 signaling pathway

**DOI:** 10.18632/aging.202909

**Published:** 2021-04-26

**Authors:** Shuangyan Lin, Mingyuan Zhao, Yanbo Lv, Genxiang Mao, Shiping Ding, Fang Peng

**Affiliations:** 1 Department of Pathology, Zhejiang Hospital, Hangzhou, Zhejiang, China; 2 Department of Geriatrics, Zhejiang Provincial Key Lab of Geriatrics, Hangzhou, Zhejiang, China; 3 Department of Cell Biology, Zhejiang University School of Medicine, Hangzhou, Zhejiang, China

**Keywords:** breast cancer, CENPU, bioinformatics analysis, miRNA, lncRNA

## Abstract

The function of centromere protein U (*CENPU*) gene in breast cancer has not been well understood. Therefore, we explored the expression profiles of *CENPU* gene in breast carcinoma to better understand the functions of this gene, as well as the relationship between *CENPU* expression and the prognosis of breast carcinoma patients. Our results indicate that *CENPU* was expressed at significantly higher levels in cancerous tissues than in normal tissues. Furthermore, *CENPU* expression correlated significantly with many clinicopathological characteristics of breast cancer. In addition, we discovered that high levels of *CENPU* expression predicted poor prognosis in patients with breast cancer. Functional investigation revealed that 180 genes exhibited co-expression with *CENPU*. Functional annotation indicated that 17 of these genes were involved in the PLK1 signaling pathway, with most of them (16/17) being expressed at significantly higher levels in malignant tissues compared with normal controls and correlating with a poor prognosis. Subsequently, we found that four miRNAs, namely hsa-miR-543, hsa-miR-495-3p, hsa-miR-485-3p, and hsa-miR-337-3p, could be regarded as potential *CENPU* expression regulators. Then, five lncRNAs were predicted to potentially bind to the four miRNAs. Combination of the results from expression, survival, correlation analysis and functional experiments analysis demonstrated the link between lncRNA GATA3-AS1/miR-495-3p/CENPU axis and prognosis of breast cancer. In conclusion, CENPU could be involved in cell cycle progression through PLK1 signaling pathway.

## INTRODUCTION

Breast cancer is common malignancy threatening female health globally. It is predicted that the incidence and mortality rate of breast cancer will rise substantially within the next 5 to 10 years [1]. As a type of aggressive malignancy with high heterogeneity, breast carcinoma has been connected with complex biological events. The occurrence of breast cancer even at a young age highlights its heterogeneity and complexity [2, 3]. Although chemotherapy, surgical resection, and radiotherapy have improved outcomes for breast cancer patients over the recent decades, the median survival of breast cancer patients with metastasis remains disappointedly low (around 24 months) [4]. Mechanisms underlying the development and progression of breast cancer remain unclear. Therefore, it is necessary to investigate the underlying molecular events and identify novel therapeutic targets and prognostic biomarkers for effective management of breast cancer.

Centromere protein U (*CENPU*) gene is localized at 4q35.1 in human genome. The gene spans a genomic DNA region of 75.8 kilobases (kb) and consists of 14 exons. The protein product of this gene has alternative names of myeloid leukemia factor 1 interacting protein (MLF1IP), Cenp-50/PBIP1, or KLIP1 [5]. According to previous studies, *CENPU* is implicated in kinetochore assembly, mitotic progression, and segregation of chromosomes [6, 7]. Our previous study demonstrated that *CENPU* downregulation might inhibit the proliferation of human breast cancer cells [8]. However, molecular mechanisms underlying that observation remain undetermined. Therefore, it is important to explore the functions of *CENPU* and its relationship with survival outcomes and pathohistological characteristics in breast carcinoma patients.

It has been demonstrated that non-coding RNAs, more specifically, microRNAs (miRNAs) and long non-coding RNAs (lncRNAs), play essential roles in tumor progression [9, 10]. Among them, miRNAs are ~22-nucleotide-long non-coding RNAs that regulate target gene expression at the post-transcriptional level [11], while lncRNAs are a class of non-coding transcripts implicated in multiple biological events, such as cell differentiation, cell growth, transcriptional and post transcriptional regulation of gene expression, and immune activation/ inactivation [12, 13]. In our current study, we also tried to identify miRNAs and lncRNAs that can regulate *CENPU* expression in breast cancer.

## RESULTS

### *CENPU* gene mutations in breast cancer

*CENPU* gene mutations in breast cancer patients were retrieved from the Catalogue of Somatic Mutations in Cancer (COSMIC) database (https://cancer.sanger.ac.uk/cosmic). Before April 7, 2020, *CENPU* gene in specimens from 37,419 patients had been sequenced, leading to the identification of 297 unique samples with *CENPU* mutations (Table 1). Among the identified mutations, 122 were point mutations, including six nonsense substitutions, 94 missense substitutions, and 22 synonymous substitutions, 3 were frameshifting insertions, and one was frameshifting deletion; No inframe deletions, inframe insertions, or complex mutations were identified (Table 1). Taken together, these data indicate a low incidence of *CENPU* gene mutation in breast carcinoma patients, implying that *CENPU* gene mutation might not be the reason for the differences in gene expression.

**Table 1:** Genetic alterations affecting CENPU in 37419 unique samples from the COSMIC database (297 unique samples with mutations in the COSMIC database).

**Genetic alteration**	**Number**	**Percentage (%)** **total number 297**	**Percentage (%)** **total number 37419**
Nonsense substitution	6	2.02	0.0002
Missense substitution	94	31.65	0.0025
Synonymous substitution	22	7.41	0.0006
Inframe insertion	0	0	0
Frameshift insertion	3	1.01	8.02E-05
Inframe deletion	0	0	0
Frameshift deletion	1	0.34	2.67E-05
Complex mutation	0	0	0
Other	30	10.10	0.0008

### Aberrant *CENPU* expression in breast carcinoma

**Figure 1:**
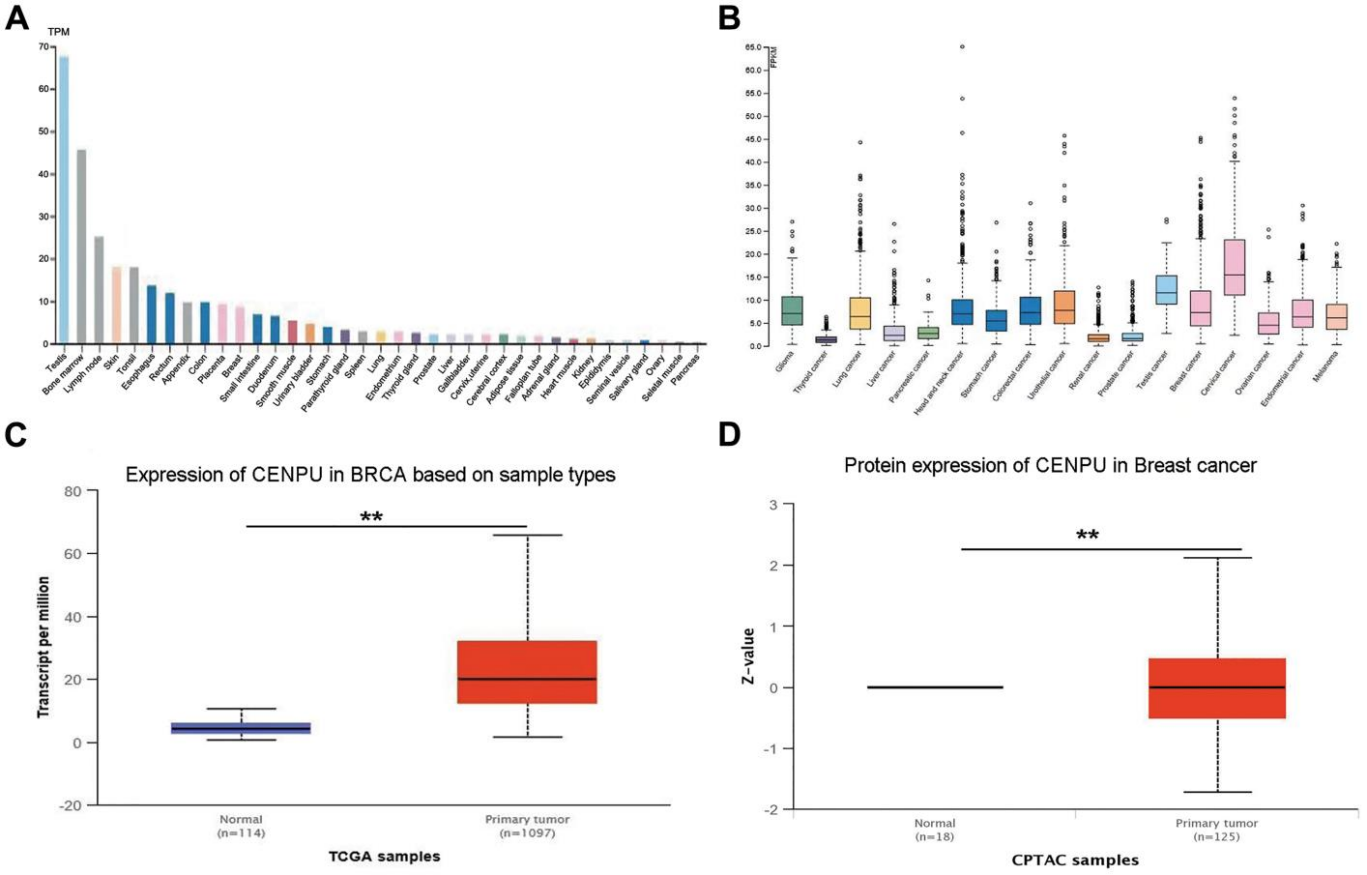
***CENPU* expression in normal and cancerous tissues.** (**A**) CENPU mRNA expression in different normal tissues from the HPA database. (**B**) CENPU mRNA expression in different cancer tissues from the HPA database. (**C**–**D**) The mRNA and protein level expression of CENPU in breast cancer compared with normal tissue by analyzing UALCAN database, ^**^P < 0.01.

An aberrant high expression of a gene in cancerous tissues is a significant indicator that the gene can be considered as a diagnostic or prognostic biomarker [14]. In view of this, based on the HPA database, we determined *CENPU* mRNA expression levels in normal and cancerous tissues. *CENPU* expression was observed in both normal and malignant breast tissues (Figure 1A–1B). Next, we conducted an analysis of *CENPU* expression in tissue samples deposited in The Cancer Genome Atlas (TCGA) and Clinical Proteomic Tumor Analysis Consortium (CPTAC) databases by using the online database UALCAN. We observed that both the mRNA and protein levels of *CENPU* were significantly higher in malignant tissues than in normal tissues (Figure 1C–1D). In addition, our previous study found significantly increased *CENPU* levels in malignant tissues than in adjacent normal breast tissues [8]. Taken together, these findings imply that *CENPU* may be a potential biomarker for breast carcinoma.

**Figure 2:**
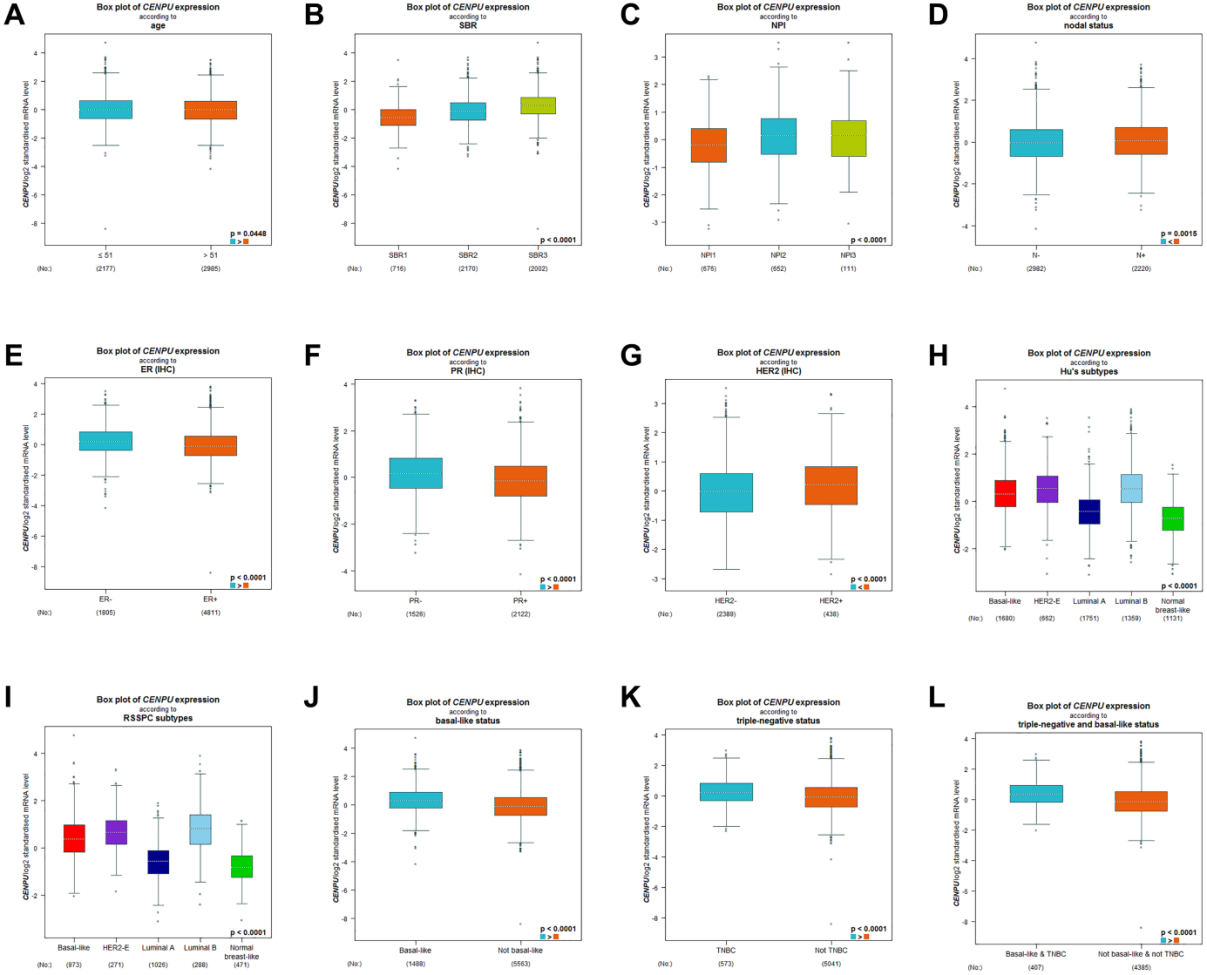
**Differences in *CENPU* expression between breast cancer patients with different clinicopathological features.** The clinicopathological features analyzed included (**A**) age, (**B**) SBR grade, (**C**) NPI score, (**D**) nodal status, (**E**) ER status, (**F**) PR status, (**G**) HER2 status, (**H**) HU's subtype, (**I**) RSSPC subtype, (**J**) basal-like status, (**K**) triple-negative status, (**L**) triple-negative and basal-like status.

Subsequently, differences in *CENPU* expression in breast carcinoma patients with different clinical and pathological parameters were determined based on bc-GenExMiner. We found significant correlation between *CENPU* expression and the age of breast cancer patients, with higher *CENPU* expression levels being observed in patients aged ≤51 years than in patients aged >51 years (Figure 2A). In addition, there were significant correlations between *CENPU* expression and Scarff-Bloom-Richardson (SBR) grade (Figure 2B) and neuropsychiatric inventory (NPI) score (Figure 2C). Furthermore, *CENPU* was markedly upregulated in patients with lymph node metastasis (Figure 2D), as well as in estrogen receptor (ER)-negative (Figure 2E), progesterone receptor (PR)-negative (Figure 2F), and human epidermal growth factor receptor 2 (HER2)-positive breast carcinoma patients (Figure 2G). The expression of *CENPU* also differed considerably among different HU’s (Figure 2H) and robust single sample predictor classification (RSSPC) subtypes (Figure 2I). Finally, we also found a significantly higher *CENPU* expression in basal-like breast carcinoma (Figure 2J), triple-negative breast carcinoma (TNBC) (Figure 2K), and basal-like & triple-negative breast triple-negative breast carcinoma (Figure 2L), compared with non-basal-like breast cancer, non-TNBC, and non-basal-like & non-triple-negative breast carcinoma, respectively.

According to eight studies included in the Oncomine database, the expression of *CENPU* was significantly higher in TNBC patients than in non-TNBC patients (fold change > 1.5, gene rank: top 10%) (Table 2). All these data suggest that *CENPU* is not only an indicator of breast cancer, but also a molecular indicator of triple-negative breast carcinoma.

**Table 2:** Elevated CENPU expression in TNBC from Oncomine database.

**Study name**	**TNBC** **patient number**	**NON-TNBC** **patient number**	**Fold change**	***P*-value (TNBC&NON-TNBC)**
Curtis breast	211	1340	2.14	7.02E-57
TCGA breast	46	250	3.04	1.84E-20
Bitner breast	39	129	2.40	4.33E-8
Hatzis breast	178	320	1.59	8.22E-17
Zhao breast	5	29	3.63	5.90E-4
Waddell breast	22	44	2.15	1.01E-5
Kao breast	32	295	1.99	1.13E-15
Richardson breast 2	18	19	2.09	5.80E-4

### Correlation of *CENPU* expression with prognosis of breast carcinoma patients

**Figure 3:**
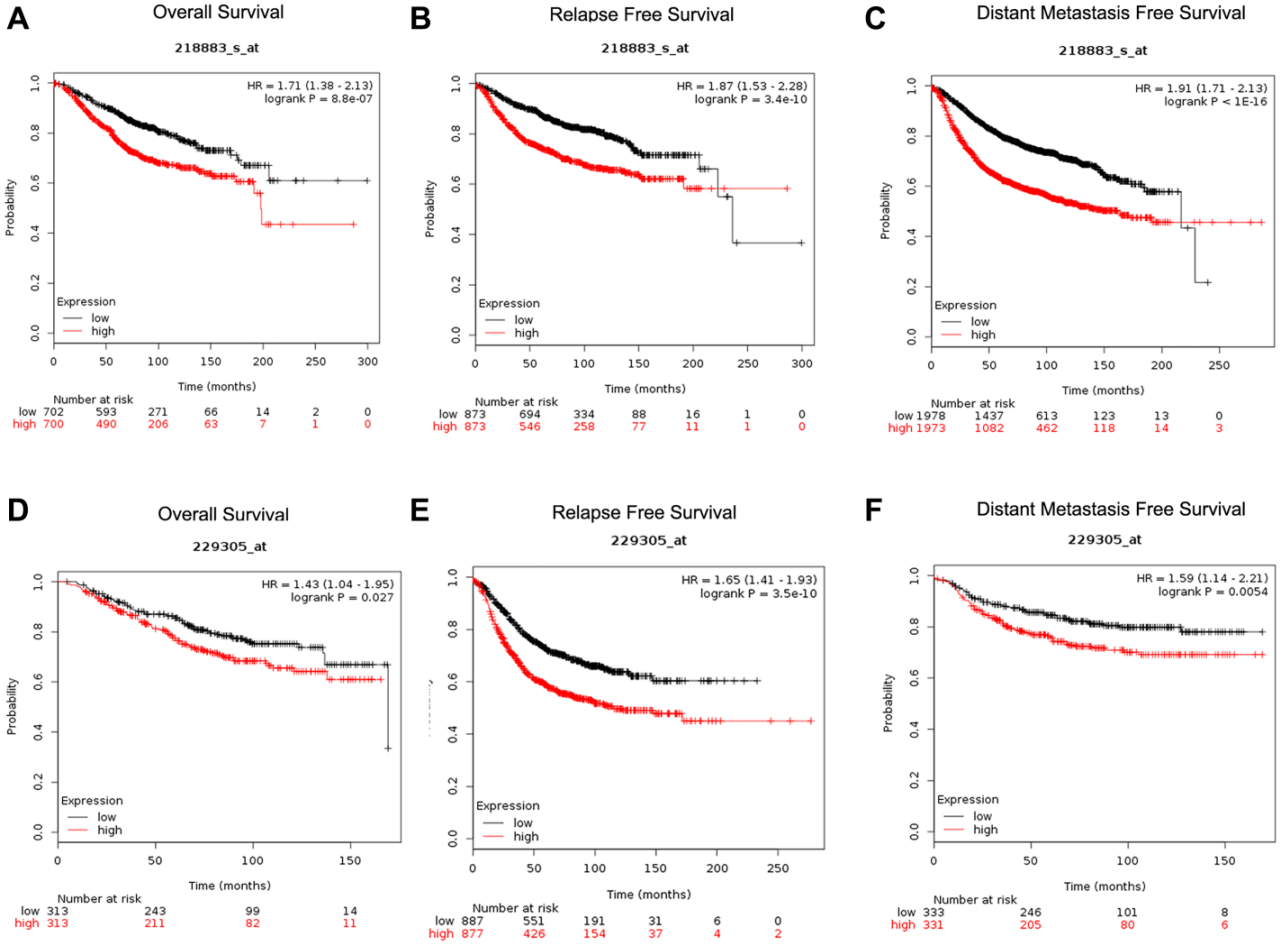
**Determination of prognostic significance of *CENPU* expression (Affymetrix IDs are valid: 218883_s_at and 229305_at) in patients with breast cancer from Kaplan-Meier plotter database.** (**A**, **D**) OS curves established according to *CENPU* expression. (**B**, **E**) RFS curves established according to *CENPU* expression. (**C**, **F**) DMFS curves established according to *CENPU* expression.

We next explored the prognostic significance of *CENPU* expression in breast cancer. Two probes (218883_s_at and 229305_at) related to *CENPU* were retrieved from the Kaplan-Meier plotter database (Figure 3). We found that *CENPU* expression significantly correlated with overall survival (OS), distant metastasis-free survival (DMFS), and relapse-free survival (RFS) in breast carcinoma patients. Specifically, high expression of CENPU was predictive of significantly poorer OS, RFS, and DMFS in both probes analyzed. Then we used the PrognoScan database to assess the correlation between *CENPU* expression and prognosis of breast carcinoma patients (Table 3). According to our analysis, *CENPU* expression level correlated significantly with OS, RFS, DMFS, disease-free survival, and disease-specific survival. Similarly, high *CENPU* expression was correlated with higher hazard ratios in breast carcinoma patients. All these data suggest that *CENPU* expression is also an indicator of prognosis of breast carcinoma patients.

**Table 3:** Relationship between CENPU expression and prognosis in breast cancer patients from PrognoScan database.

**Dataset**	**Endpoint**	**Probe ID**	**Number**	**Cox *p*-value**	**HR [95% CI]**
GSE12276	Relapse Free Survival	218883_s_at	204	0.04116360	1.26 [1.01–1.57]
GSE6532-GPL570	Distant Metastasis Free Survival	229304_s_at	87	0.00262684	2.34 [1.34–4.07]
GSE6532-GPL570	Relapse Free Survival	229305_at	87	0.01880120	1.59 [1.08–2.34]
GSE6532-GPL570	Distant Metastasis Free Survival	229305_at	87	0.01880120	1.59 [1.08–2.34]
GSE6532-GPL570	Relapse Free Survival	229304_s_at	87	0.00262684	2.34 [1.34–4.07]
GSE12093	Distant Metastasis Free Survival	218883_s_at	136	0.01926210	2.30 [1.15–4.62]
GSE11121	Distant Metastasis Free Survival	218883_s_at	200	0.00213651	2.25 [1.34–3.78]
GSE2034	Distant Metastasis Free Survival	218883_s_at	286	0.00041530	1.86 [1.32–2.62]
GSE1456-GPL96	Overall Survival	218883_s_at	159	0.00015424	2.55 [1.57–4.15]
GSE1456-GPL96	Relapse Free Survival	218883_s_at	159	0.00006250	2.71 [1.66–4.42]
GSE1456-GPL96	Disease Specific Survival	218883_s_at	159	0.00034302	2.84 [1.60–5.02]
GSE1456-GPL97	Overall Survival	229305_at	159	0.00217077	2.64 [1.42–4.90]
GSE1456-GPL97	Relapse Free Survival	229305_at	159	0.01827860	2.11 [1.13–3.92]
GSE1456-GPL97	Disease Specific Survival	229305_at	159	0.02115490	2.36 [1.14–4.90]
GSE7378	Disease Free Survival	218883_s_at	54	0.02591300	2.40 [1.11–5.18]
GSE3494-GPL96	Disease Specific Survival	218883_s_at	236	0.00078752	2.25 [1.40–3.62]
GSE3494-GPL97	Disease Specific Survival	229305_at	236	0.01304810	2.37 [1.20–4.69]
GSE3494-GPL97	Disease Specific Survival	229304_s_at	236	0.00399709	4.41 [1.61–12.10]
GSE4922-GPL96	Disease Free Survival	218883_s_at	249	0.00051062	1.98 [1.35–2.90]
GSE4922-GPL97	Disease Free Survival	229305_at	249	0.00044163	2.67 [1.54–4.63]
GSE4922-GPL97	Disease Free Survival	229304_s_at	249	0.00811801	2.91 [1.32–6.42]
GSE2990	Relapse Free Survival	218883_s_at	125	0.00791330	1.49 [1.11–2.01]
GSE2990	Distant Metastasis Free Survival	218883_s_at	54	0.02650860	1.91 [1.08–3.38]
GSE2990	Relapse Free Survival	218883_s_at	62	0.01579130	1.74 [1.11–2.73]
GSE7390	Relapse Free Survival	218883_s_at	198	0.02837110	1.30 [1.03–1.65]
GSE7390	Distant Metastasis Free Survival	218883_s_at	198	0.00208122	1.58 [1.18–2.11]
GSE7390	Overall Survival	218883_s_at	198	0.00025020	1.80 [1.31–2.46]

### The involvement of *CENPU* in the PLK1 signaling pathway

**Figure 4:**
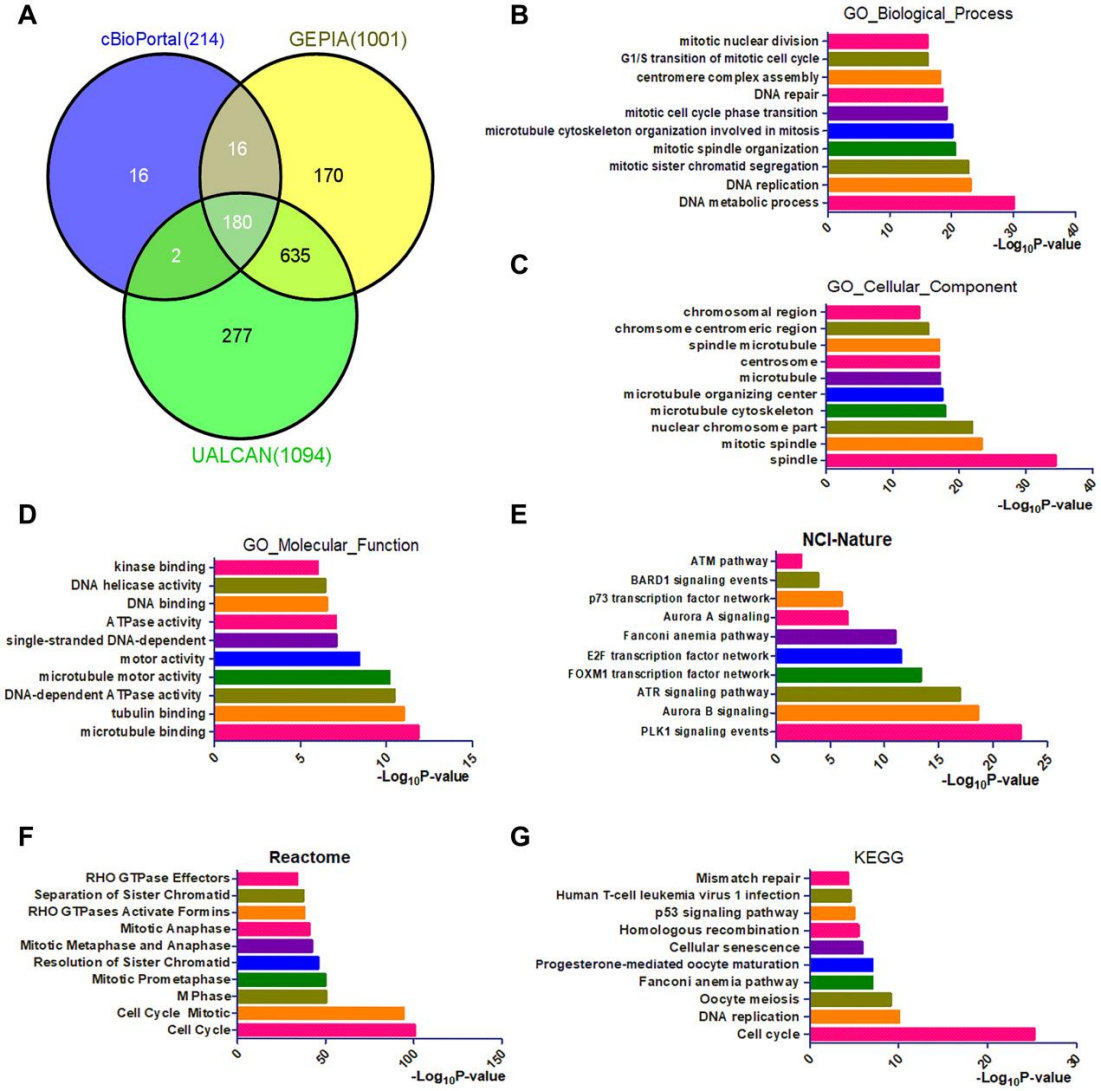
**Identification and functional analysis of genes that exhibited co-expression with *CENPU*.** (**A**) The Venn diagram of CENPU’s co-expressed genes from UALCAN, GEPIA, and cBioPortal databases. (**B**–**D**) GO functional annotation (biological process, cellular component, and molecular function) for 180 co-expressed genes of CENPU. (**E**–**G**) Pathway (NCI-Nature, Reactome, and KEGG) enrichment analysis for these 180 co-expressed genes of CENPU.

To better understand the functions of *CENPU*, we utilized three databases (cBioPortal, GEPIA, and UALCAN) to identify genes that exhibited co-expression with *CENPU*. We found that the number of genes co-expressed with *CENPU* was 214, 1001, and 1094, as revealed by the cBioPortal, GEPIA, and UALCAN databases, respectively (Figure 4A). Venn diagram showed that the number of genes exhibited co-expression with *CENPU* was 180. To gain a better understanding of these genes, we conducted gene ontology (GO) annotation and pathway enrichment analyses with the Enrichr database (Figure 4B–4G). For functional annotation, three categories of GO term were analyzed, including biological process, cellular component, and molecular function. For pathway enrichment, cell signaling pathways included in the NCI-Nature Pathway, Reactome Pathway, and Kyoto Encyclopedia of Genes and Genomes (KEGG) Pathway were analyzed. As presented in Figure 4B–4D, the top two GO terms enriched were DNA metabolic process and DNA replication in the GO category of biological process, spindle and mitotic spindle in the category of cellular component, and microtubule binding and tubulin binding in the category of molecular function. As shown in Figure 4E–4G, the most enriched pathways in the three GO categories were cell cycle-related. It is noteworthy that the PLK1 signaling pathway was the most enriched signaling cascade in the NCI-Nature Pathway.

**Figure 5:**
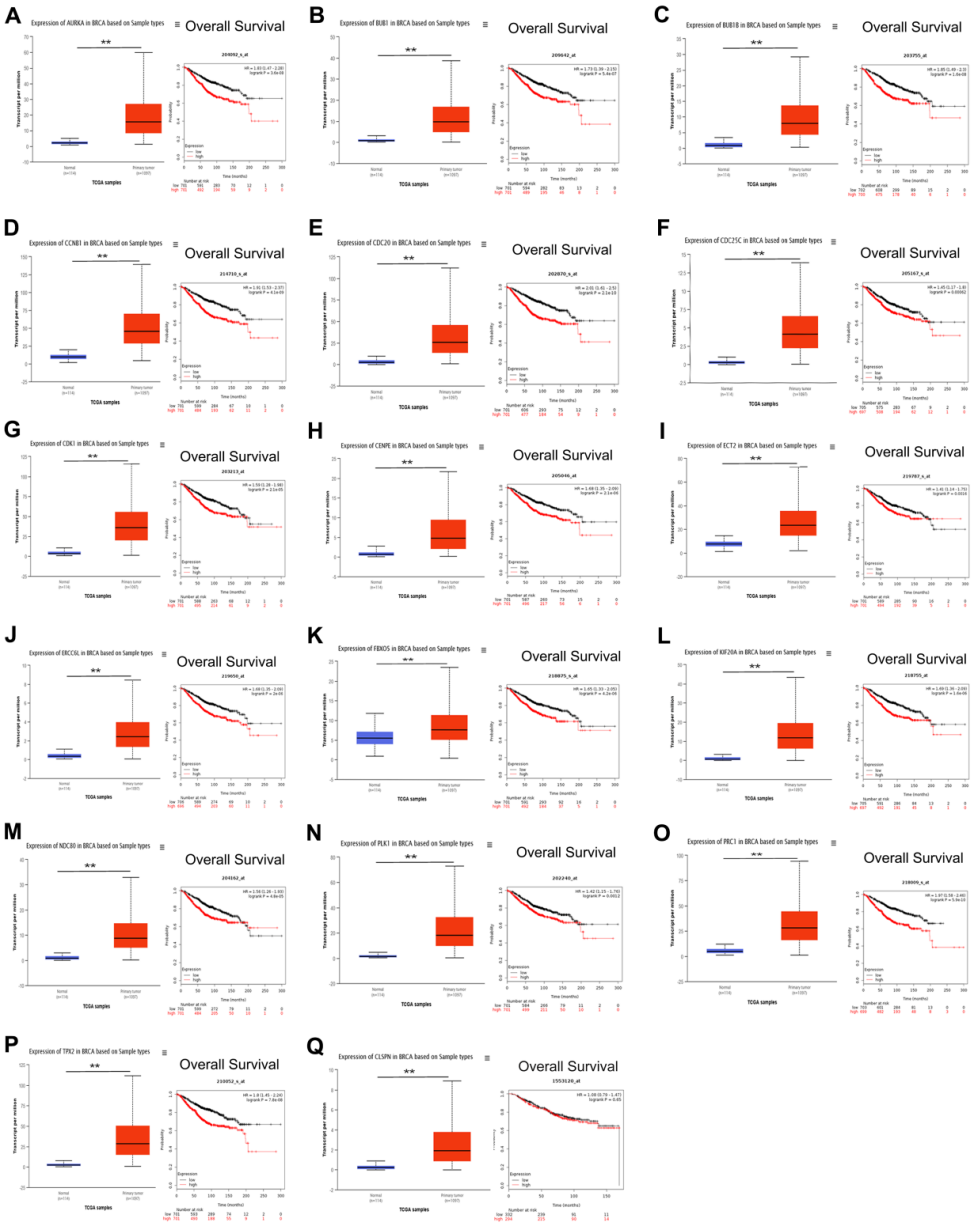
(**A**–**Q**) Prognostic value of the 17 genes that exhibited co-expression with *CENPU* and are involved in the PLK1 pathway in breast cancer. ^**^P < 0.01.

**Figure 6:**
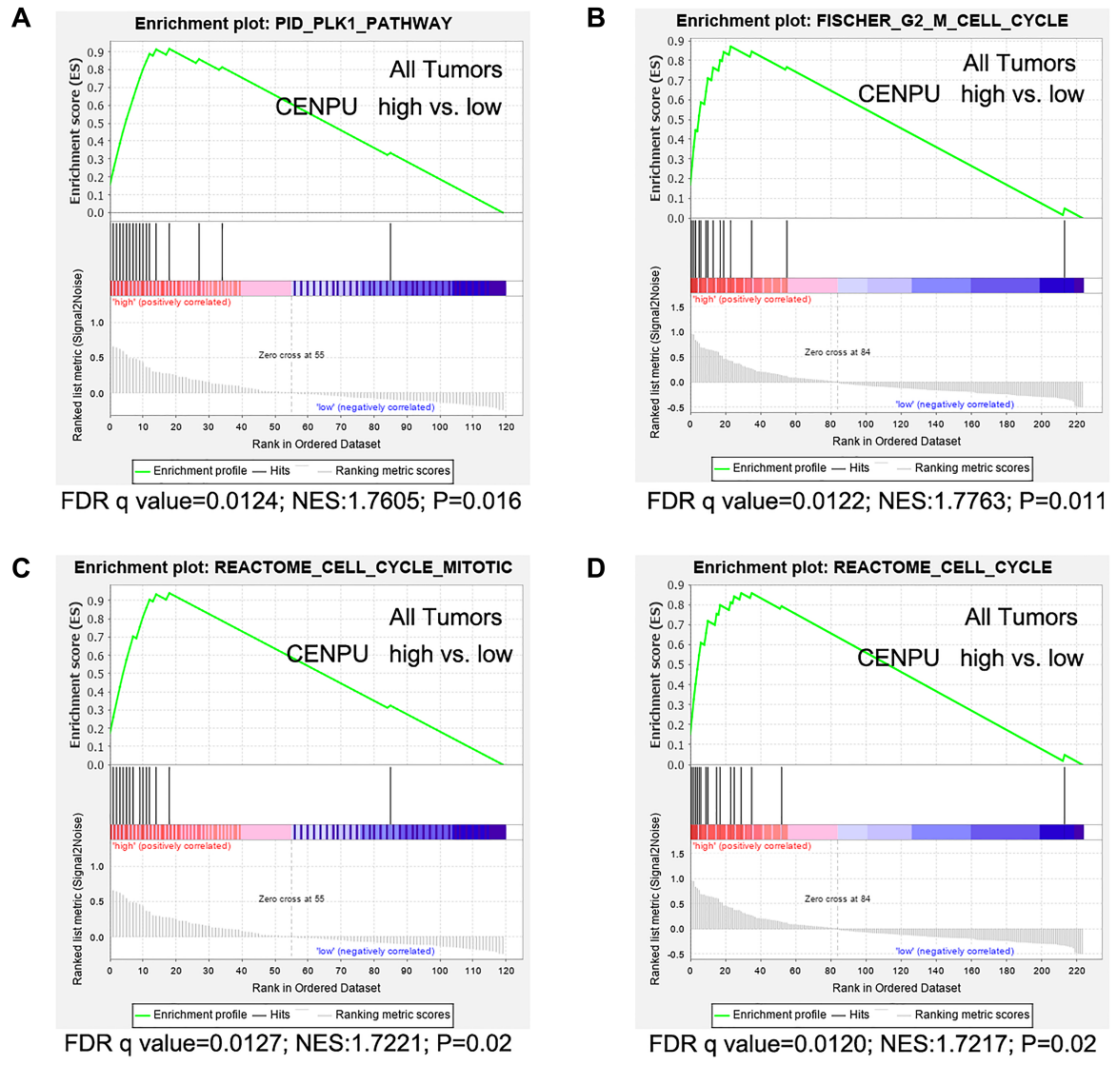
**Gene set enrichment analyses were performed using gene expression data of 226 African-American women with triple negative breast cancer (TNBC).** (**A**) Genes are enriched in PLK1 pathway. (**B**) Genes are enriched in Fisher G2-M cell cycle. (**C**) Genes are enriched in the Reactome cell cycle mitotic. (**D**) Genes are enriched in Reactome cell cycle. False discovery rate (FDR) q value, normalized enrichment score (NES), and *P* values are shown, vs: versus.

Since the PLK1 signaling pathway is associated with cell cycle, we next focused on PLK1 signaling-related events. In addition to *CENPU*, there were 17 other genes enriched in the PLK1 signaling cascade (Table 4). The correlation coefficients between these genes and *CENPU* were in the range of 0.32-0.69 according to the cBioPortal, GEPIA, and UALCAN databases. Next, we compared the expression levels of these 17 genes in breast carcinoma tissues with those in normal controls by utilizing the UALCAN database (Figure 5). All these genes showed a significantly higher expression in malignant tissues than in their normal counterparts. We then determined the prognostic significance of these genes in breast cancer by utilizing the Kaplan-Meier plotter database and found that high expression levels of 16 genes were associated with poor OS (Figure 5). These results were similar to those observed on *CENPU*. Gene Set Enrichment Analysis (GSEA) showed that the 17 genes co-expressed with *CENPU* were enriched in PLK1 pathway, G2/M cell cycle, cell cycle mitotic, and cell cycle from reactome in TNBC along with high expression of CENPU (Figure 6A–6D). All these data imply that *CENPU* may be involved in cell cycle progression through the PLK1 signaling pathway. The details of PLK1 pathway enriched genes in TNBC patients with CENPU high vs. CENPU low was shown in the Supplementary Table 1.

**Table 4:** The correlation between PLK1 signaling-related genes and CENPU from cBioPortal, GEPIA and UALCAN databases.

**Gene name**	**R (cBioPortal)**	**R (GEPIA)**	**R (UALCAN)**
AURKA	0.66	0.51	0.51
BUB1	0.66	0.51	0.53
BUB1B	0.68	0.62	0.62
CCNB1	0.68	0.55	0.56
CDC20	0.57	0.32	0.34
CDC25C	0.67	0.62	0.61
CDK1	0.69	0.51	0.51
CENPE	0.68	0.63	0.63
ECT2	0.54	0.56	0.56
ERCC6L	0.63	0.53	0.53
FBXO5	0.53	0.49	0.49
KIF20A	0.64	0.54	0.54
NDC80	0.65	0.49	0.49
PLK1	0.61	0.48	0.47
PRC1	0.68	0.54	0.56
TPX2	0.68	0.54	0.54
CLSPN	0.55	0.53	0.53

### Identification of key miRNAs that can potentially regulate *CENPU* expression

**Figure 7:**
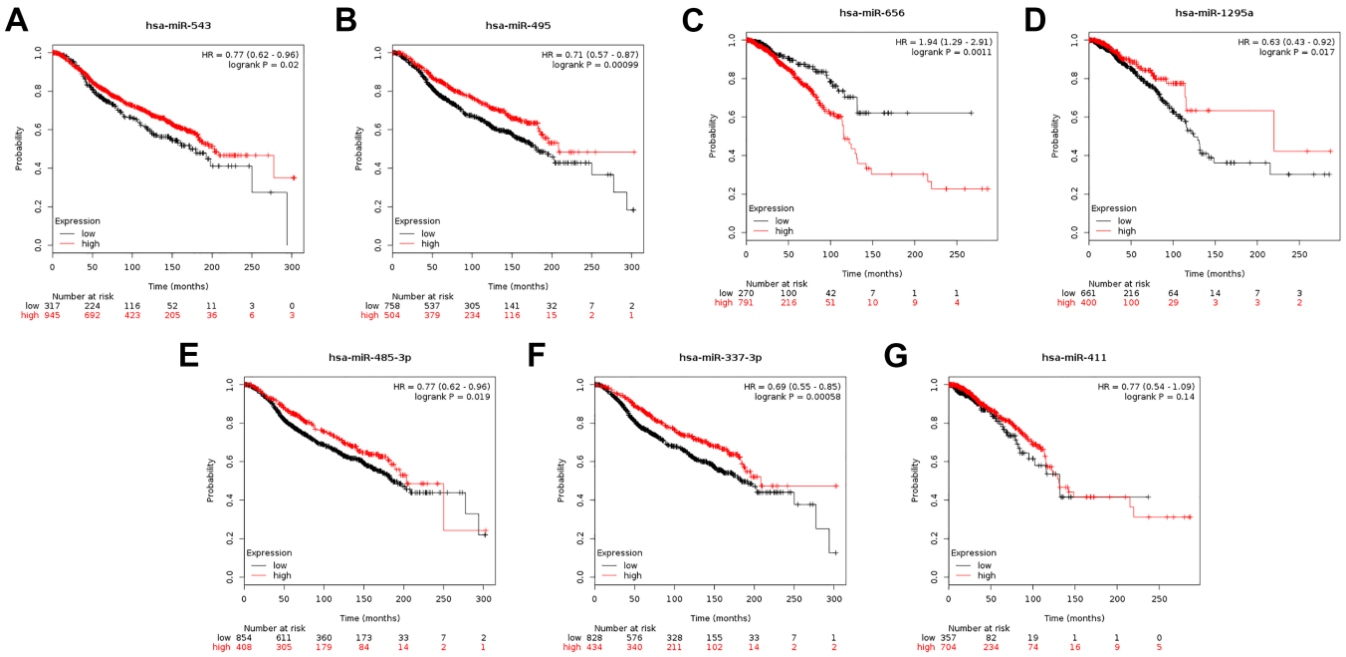
(**A**–**G**) Prognostic significance of the seven miRNAs that can potentially regulate *CENPU* expression in breast cancer analyzed by Kaplan-Meier plotter database.

MicroRNAs are endogenous RNAs that modulate expression of target genes post-transcriptionally [11]. Based on the functions of *CENPU* in breast cancer, we used the starBase database to predict miRNAs that can potentially regulate *CENPU* expression and identified eight such miRNAs, namely hsa-miR-543, hsa-miR-495-3p, hsa-miR-493-3p, hsa-miR-656-3p, hsa-miR-1295a, hsa-miR-485-3p, hsa-miR-337-3p, and hsa-miR-411-5p (Table 5). Furthermore, we assessed correlations between the expression levels of these predicted miRNAs and *CENPU* expression using the OncomiR database. Seven miRNAs, including hsa-miR-543, hsa-miR-495-3p, hsa-miR-656-3p, hsa-miR-1295a, hsa-miR-485-3p, hsa-miR-337-3p, and hsa-miR-411-5p, were found downregulated in malignant tissues compared with normal controls (Table 6). Based on the Kaplan-Meier plotter database, we subsequently evaluated prognostic significance of these miRNAs in breast cancer (Figure 7) and found that higher expression levels of five miRNAs (namely hsa-miR-543, hsa-miR-495-3p, hsa-miR-1295a, hsa-miR-485-3p, and hsa-miR-337-3p) were predictive of a more favorable prognosis. It is well accepted that the expression of a miRNA is negatively associated with that of its target mRNA [15]. Given that larger correlation coefficients indicate stronger correlations, four miRNAs (namely hsa-miR-543, hsa-miR-495-3p, hsa-miR-485-3p, and hsa-miR-337-3p) with correlation coefficient absolute values above 0.1 were regarded as the “key miRNAs”.

**Table 5:** The correlation between predicted miRNA and CENPU.

**Predicted miRNA**	**R**	***P*-value**
hsa-miR-543	–0.109	3.30e-4
hsa-miR-495-3p	–0.107	4.30e-4
hsa-miR-493-3p	–0.070	2.18e-2
hsa-miR-656-3p	–0.164	5.91e-8
hsa-miR-1295a	–0.062	4.23e-2
hsa-miR-485-3p	–0.105	5.03e-4
hsa-miR-337-3p	–0.173	9.41e-9
hsa-miR-411-5p	–0.149	7.79e-7

**Table 6:** The expression of potential miRNAs in breast cancer from OncomiR database.

**miRNA Name**	***T*-test *P*-value**	***T*-test FDR**	**Downregulated**	**Tumor log2** **mean expression**	**Normal log2** **mean expression**
hsa-miR-543	5.77e-06	1.77e-05	Tumor	0.10	0.47
hsa-miR-495-3p	5.49e-20	6.89e-19	Tumor	2.43	3.98
hsa-miR-656-3p	4.58e-08	1.69e-07	Tumor	0.09	0.51
hsa-miR-1295a	5.05e-10	2.33e-09	Tumor	0.35	1.12
hsa-miR-485-3p	3.78e-07	1.31e-06	Tumor	1.83	2.57
hsa-miR-337-3p	4.52e-24	1.02e-22	Tumor	5.08	6.82
hsa-miR-411-5p	4.81e-16	3.88e-15	Tumor	1.96	3.14

### Identification of key lncRNAs that can potentially regulate the key miRNAs

**Figure 8:**
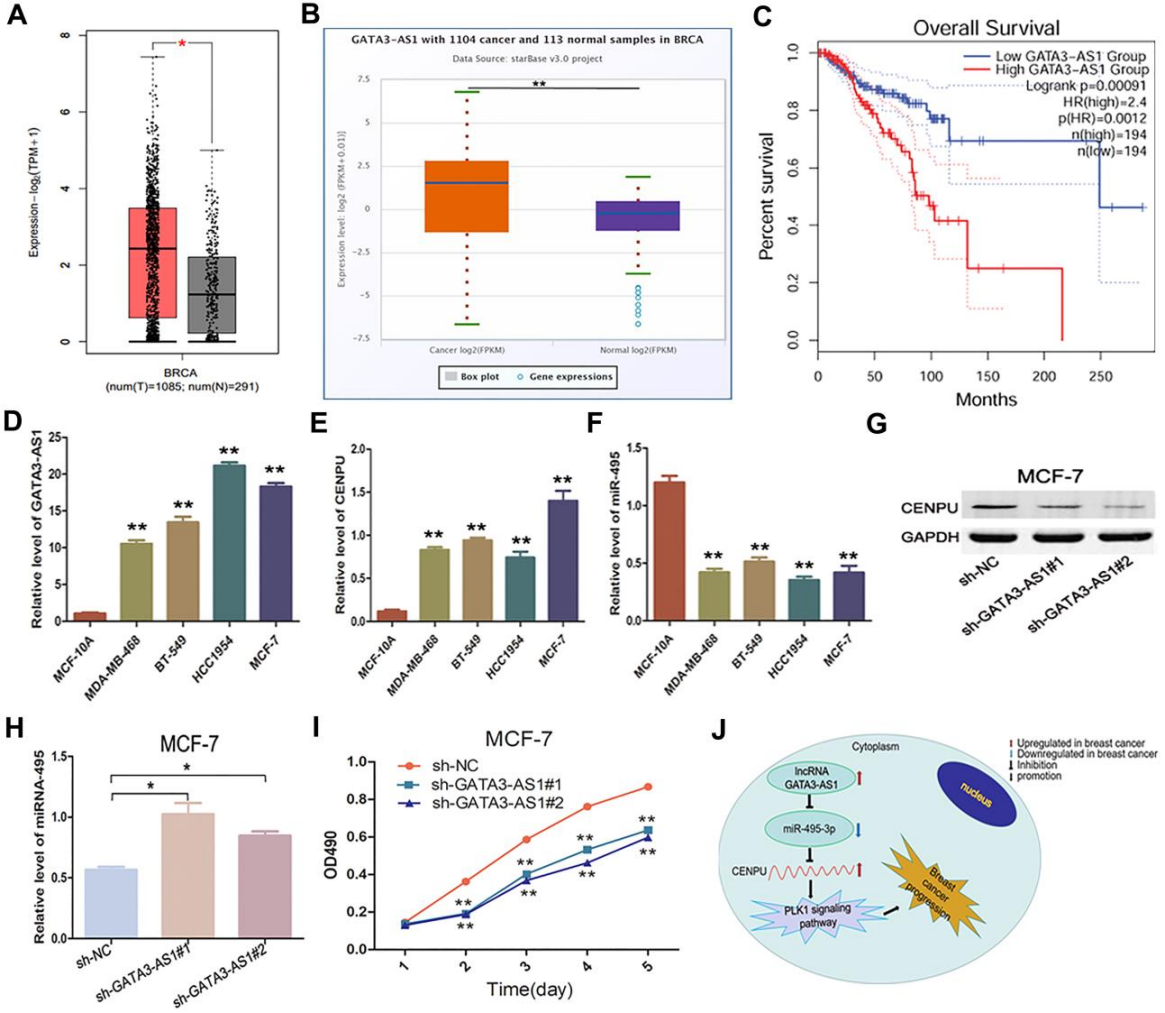
**Expression and biological significance of the lncRNA GATA3-AS1 in breast carcinoma.** (**A**) Expression levels of GATA3-AS1 in breast carcinoma as determined with GEPIA. (**B**) Expression levels of GATA3-AS1 in breast cancer as determined with the starBase database. (**C**) Prognostic value of GATA3-AS1 in breast cancer as determined with GEPIA. (**D**–**F**) Relative expression levels of GATA3-AS1, *CENPU*, and miR-495-3p in MDA-MB-468, BT-549, HCC1954, MCF-7, and MCF-10A cell lines as determined by qRT-PCR. (**G**) Western blotting analysis of CENPU protein levels in MCF-7 cells after the cells were transfected with sh-NC, sh-GATA3-AS1#1, sh-GATA3-AS1#2. (**H**) Quantitative RT-PCR analysis of miR-495-3p levels in MCF-7 cells after the cells were transfected with sh-NC, sh-GATA3-AS1#1, sh-GATA3-AS1#2. (**I**) Proliferation of MCF-7 cells after the cells were transfected with sh-NC, sh-GATA3-AS1#1, sh-GATA3-AS1#2 by MTT assay. (**J**) The identified lncRNA-miRNA-mRNA axis that can predict the prognosis of breast cancer patients. ^**^P < 0.01, ^*^P < 0.05.

Growing evidence has suggested that lncRNAs can function as ceRNAs to interact with mRNA by competing for shared miRNA [16]. Based on this principle, we used the starBase database to identify lncRNAs that can potentially bind with the four above-mentioned key miRNAs. Five such lncRNAs were eventually identified (Table 7). Based on the aforementioned ceRNA theory, two lncRNAs with correlation coefficient absolute values above 0.1 (GATA3-AS1 and PAXIP1-AS1) were selected for subsequent analyses. Furthermore, by using the GEPIA and starBase databases, we found that only GATA3-AS1 exhibited significantly higher levels in malignant samples than in their normal counterparts (Figure 8A–8B). Therefore, we next tried to explore the correlation between GATA3-AS1 expression and survival of breast cancer patients. Our results indicate that higher expression levels of GATA3-AS1 correlate with worse prognosis (Figure 8C). To further verify this finding *in vitro*, we carried out loss-of-function analyses in breast carcinoma cell lines. Quantitative reverse-transcription PCR (qRT-PCR) assays demonstrated that both GATA3-AS1 and *CENPU* were markedly overexpressed in MDA-MB-468, BT-549, HCC1954, and MCF-7 (three breast cancer cell lines) than in MCF-10A cells (normal human breast epithelial cells), while the expression of miR-495-3p was lower in breast cancer cell lines than in normal breast epithelial cells (Figure 8D–8F). By silencing GATA3-AS1, *CENPU* was downregulated (Figure 8G) and miR-495 was upregulated (Figure 8H) in MCF-7 cells. Additionally, the proliferation of MCF-7 cells was suppressed (Figure 8I). Therefore, GATA3-AS1 was defined as a key lncRNA. Combined with the results obtained from the expression analysis, survival analysis, and correlation analyses, these functional experiments demonstrate a link between the lncRNA GATA3-AS1/miR-495-3p/CENPU axis and the prognosis of breast cancer patients (Figure 8J).

**Table 7:** The correlation between miRNA-lncRNA pairs in breast cancer identified by starBase database.

**miRNA**	**lncRNA**	**R**	***P*-value**
has-miR-543	NORAD	–0.082	6.65e-3
has-miR-495-3p	NORAD	–0.060	4.66e-2
	JPX	–0.098	1.28e-3
	PURPL	–0.061	4.35e-2
	GATA3-AS1	–0.126	3.26e-5
hsa-miR-485-3p	PAXIP1-AS1	–0.178	3.73e-9

## DISCUSSION

Despite great efforts have been invested in breast cancer biology during the past decades, the disease still poses a serious threat to the global public health. ER^-^/PR^-^/HER2^-^ breast cancer, also defined as triple-negative breast cancer (TNBC), is a most common and life-threatening breast cancer subtype with the worst prognosis [17]. However, the molecular mechanisms underlying TNBC tumorigenesis are still elusive. Therefore, we tried to find a new molecular target for breast cancer treatment in this study. We first analyzed *CENPU* gene mutations in breast carcinoma patients and found a relatively low incidence of *CENPU* gene mutation, implying that *CENPU* gene mutation might not be the key reason for the differences in its mRNA level. Then we analyzed the expression profile of *CENPU* gene based on the HPA database. Our data indicated that the expression of *CENPU* was significantly higher in malignant tissues than in normal tissues. Also, *CENPU* expression significantly correlated with clinical and pathological features of breast carcinoma patients, especially patients with TNBC, implying that the expression of *CENPU* was closely linked to progression of breast carcinoma. Next, based on the PrognoScan database, we explored the prognostic significance of *CENPU* expression in breast carcinoma and found that higher *CENPU* expression levels were predictive of a poorer prognosis. Taken together, these data imply that *CENPU* is a molecular indicator for breast carcinoma, especially for TNBC.

Next, we explored the mechanism underlying *CENPU*’s prognostic value in breast cancer. Co-expression analysis identified 180 genes co-expressed with *CENPU*. Functional annotation demonstrated that these genes were significantly enriched in the PLK1 signaling pathway. Among these genes, 17 were directly involved in the PLK1 signaling pathway. We then observed that most PLK1 signaling pathway-related genes were aberrantly upregulated in breast cancer, and that high expression of most of these genes closely correlated with a poor prognosis. Combined with our previous findings, we propose that *CENPU* is involved in the PLK1 signaling pathway.

Subsequently, we aimed to explore how *CENPU* was regulated in breast cancer. It was previously reported that miRNAs are natural inhibitors of oncogenes and suppressors of tumorigenesis [18]. In this study, we identified four miRNAs, namely hsa-miR-543, hsa-miR-495-3p hsa-miR-485-3p, and hsa-miR-337-3p, as key miRNAs that can potentially regulate *CENPU* expression. Our subsequent lncRNA analysis identified GATA3-AS1, which can bind with has-miR-495-3p, as a key lncRNA. Finally, correlation analysis revealed that only the lncRNA GATA3-AS1/miR-495-3p/CENPU axis conformed to the ceRNA theory. Functional analyses were then carried out to investigate the molecular events downstream of GATA3-AS1 and the relationships between *CENPU*, miRNA-495, and GATA3-AS1. The RT-qPCR results revealed that GATA3-AS1 exhibited significantly higher expression levels in breast cancer cell lines than in normal control. In addition, the expression of GATA3-AS1 was positively correlated with *CENPU* expression and negatively correlated with that of miRNA-495, which is consistent with our bioinformatics analysis results. Finally, in terms of biological function, GATA3-AS1 knockdown inhibited the proliferation of breast cancer cells. These results suggest that GATA3-AS1 plays a crucial role in tumorigenesis of breast cancer.

In conclusion, using integrated bioinformatics analysis, we found a novel lncRNA-miRNA-mRNA axis of high prognostic value in breast carcinoma. More experiments and large-scale clinical trials are required in the future to further verify our results.

## MATERIALS AND METHODS

### Analysis based on the COSMIC database

COSMIC is an online database containing information about all types of genomic alteration in human malignancies [19]. Using COSMIC version 91, we summarized gene mutations that can potentially affect *CENPU* expression. All the data were retrieved on April 7, 2020.

### Analysis based on the HPA database

The HPA database is dedicated to providing immunohistochemical data regarding the expression and distribution of 24,000 identified human proteins in multiple types of cells, cell lines and normal or cancerous tissues [20]. Based on this database, we determined *CENPU* expression in various normal and cancerous tissues.

### Correlations between *CENPU* levels and clinical and pathohistological characteristics in breast carcinoma

Differences in *CENPU* expression in breast carcinoma patients with diverse clinical and pathological parameters were investigated based on bc-GenExMiner [21], a database exhibiting gene expression profiles in breast cancer patients. *CENPU* expression was examined in patients with different ages, SBR grades, Nottingham prognostic index (NPI) scores, lymph node statuses, ER statuses, PR statuses, HER2 statuses, HU’s subtypes, RSSPC subtypes, basal-like statuses, and triple-negative statuses.

### Analysis based on the Oncomine database

Oncomine is an online discovery platform providing transcriptomic information based on 715 datasets obtained from 86,733 cancerous and normal tissue samples [22]. Using Oncomine version 4.5, gene expression profiles were analyzed and compared between different types and subtypes of malignancy, among cancer patients with different clinicopathological features, and between cancerous and normal tissues.

### Analyses based on the Kaplan–Meier plotter and prognoScan databases

Kaplan-Meier plotter is a database that provides information about the correlation between expression of a specific gene and survival of cancer patients [23]. PrognoScan is a newly established base that collects meta-analyses of prognostic significance of different genes in various human malignancies [24]. We previously explored the expression of *CENPU* in cancerous and normal tissues of breast carcinoma patients based on the online database UALCAN [25]. In this research, we investigated the correlations between *CENPU* expression and OS, RFS, DMFS, or post-progression survival in those patients by utilizing the Kaplan-Meier plotter database. In addition, genes exhibited co-expression with *CENPU* were identified based on the UALCAN database, and their prognostic values were predicted based on the Kaplan-Meier plotter database.

### Analyses of genes co-expressed with *CENPU*

We characterized genes that exhibited co-expression with *CENPU* using the cBioPortal [26], GEPIA [27], and UALCAN databases. To characterize the functions of these genes, GO annotation and pathway enrichment analyses were carried out based on the Enrichr database [28].

To obtain a better understanding of the functions of these genes, we retrieved raw microarray data from the gene expression omnibus (GEO) database. The dataset GSE142102 obtained based on platform GPL17692 from a cohort of 226 African-American female TNBC patients was used. Subsequently, these 226 samples were categorized into two groups depending on the level of *CENPU* expression (high vs. low). GSEA [29] was then carried out utilizing the optimal cut-off expression values established for event-free survival (EFS). We enriched gene sets obtained from the MSigDB “Curated” gene set collection (https://www.gsea-msigdb.org/gsea/msigdb/genesets.jsp) in both the CENPU-high and the CENPU-low groups.

### Analysis of key miRNAs and lncRNAs

We predicted miRNAs and lncRNAs that can potentially regulate *CENPU* expression by using starBase, a comprehensive online resource for prediction of microRNAs and lncRNAs [30]. Then we validated the functions and prognostic values of the predicted genes by using Gene Expression Profiling Interactive Analysis (GEPIA) (http://gepia.cancer-pku.cn/detail.php), a novel online server for analysis of RNA-seq data deposited in TCGA and GTEx.

### Cell culture and cell transfection

Human breast cancer cell lines MDA-MB-468, BT-549, HCC1954, and MCF-7, and a normal breast epithelial cell line MCF-10 A were purchased from ATCC and maintained in Dulbecco's Modified Eagle Medium (Thermo Fisher Scientific) containing 10% fetal bovine serum (HyClone) and 1% penicillin-streptomycin solution under the conditions of 37°C, 5% CO_2_. The shRNAs targeting GATA3-AS1 and negative control (NC)-shRNAs were synthesized by GenePharma and were transfected into MCF-7 cells by utilizing the Lipofectamine 2000 transfection kit (Invitrogen).

### Cell viability assay

Equal amounts (2 × 10^3^) of cells were seeded into each well of five 96-well plates. The cells were cultured for five consecutive days and added with 20 μL of 3-(4,5-dimethylthiazol-2-yl)-2,5-diphenyltetrazolium bromide (MTT, final concentration 5 mg/mL). Afterwards, the cells were maintained at 37°C, 5% CO_2_ for another 4 hours, and the MTT solution was removed. The 490-nm optical absorption value of each well was obtained and cell grow curves were established accordingly by using the Graphpad Prism 5.0 software.

### Quantitative reverse transcription PCR (qRT-PCR)

Total RNA was extracted from cells using the TRIzol reagent (Invitrogen) as per the manufacturer’s protocol. The RNA was then reverse-transcribed into cDNA using a cDNA synthesis kit (Promega). After that, relative expression levels of target genes were quantified by qRT-PCR assays with the SYBR_Premix ExTaq II kit (Toyobo) on an Applied Biosystems 7500 Real-Time PCR platform (Applied Biosystems). The following primers were used in this experiment: GATA3-AS1 F: 5′-TTGTTCCCTCTTCGCTCCT-3′ and GATA3-AS1 R: 5′-TTGTTCCTTCACCGCATG-3′; CENPU F: 5′-ATGAACTGCTTCGGTTAGAGC-3′ and CENPU R: 5′-TATTTCGCAGATGGCTTTCGG-3′; and miR-495 F: 5′-ACACTCCAGCT GGGGAAGTTGCCCATGTT-3′ and miR-495 R: 5′-CTCAACT GGTGTCGTGGA-3′; The 2^-ΔΔCt^ method was utilized for calculation of relative fold changes of target genes, with the expression level of *GAPDH* gene as the internal reference.

### Immunoblotting

MCF-7 cell lysates were prepared and electrophoresed on 10% sodium dodecyl sulfate polyacrylamide gel for separation of proteins, which were then electro-blotted onto polyvinyl fluoride membranes (Millipore). After being incubated in 5% non-fat dry milk [dissolved in Tris-buffered saline Tween (TBST)] for 1 h at ambient temperature, the membranes were probed with primary antibodies raised against our target proteins at 4°C overnight. After being rinsed with TBST for three times, the membranes were subjected to incubation in horseradish peroxidase (HRP)-conjugated secondary antibodies for 1 h at ambient temperature. The protein bands were eventually developed by using the Enhanced Chemiluminescence Western Blotting Substrate kit (Pierce). The primary antibodies used in this study included rabbit anti-CENPU (diluted 500-fold; Abcam, ab117078) and mouse anti-GAPDH (diluted 2000-fold; Santa Cruz, sc-32233); the secondary antibodies used in this study included goat anti-rabbit IgG (diluted 2000-fold; Santa Cruz, sc-2004) and goat anti-mouse IgG (diluted 2000-fold; Santa Cruz, sc-2005).

### Statistical analysis

At least three replicates were carried out for each experiment, and the results were analyzed by the GraphPad prism 5.0 software (GraphPad Software, Inc.). Quantitative data are displayed as mean ± S.D. We performed Student’s *t*-test to evaluate the statistical significance of intergroup differences, and a *P* value < 0.05 was regarded as statistically significant.

## Supplementary Materials

Supplementary Table 1
