# Supplementary material for: The lncRNA GATA3-AS1/miR-495-3p/CENPU axis predicts poor prognosis of breast cancer via the PLK1 signaling pathway
Source: Aging (Albany NY). 2021 Apr 26;13(10):13663–79. doi: 10.18632/aging.202909 (PMC8202843; doi:10.18632/aging.202909)
Supplement: Supplementary Table 1 [file aging-13-202909-s001.pdf]

SUPPLEMENTARY TABLE

Supplementary Table 1. GSEA details of PLK1 pathway enriched genes in TNBC patients with CENPU high vs. CENPU low.

Gene Name	Rank Metric Score	Running ES	Core Enrichment
PLK1	1.673963785	0.14937250	Yes
TPX2	0.954416513	0.23453778	Yes
CDC20	0.941986740	0.31859392	Yes
CENPE	0.829827487	0.39264175	Yes
NDC80	0.765826583	0.45612425	Yes
CCNB1	0.685156107	0.51726264	Yes
BUB1	0.649955809	0.56555130	Yes
KIF20A	0.647423625	0.62332270	Yes
ECT2	0.623647332	0.66926384	Yes
CENPU	0.608254910	0.71868575	Yes
CDK1	0.524208784	0.76060800	Yes
PRC1	0.456795961	0.79651480	Yes
BUB1B	0.417752266	0.81922895	Yes
ERCC6L	0.369548827	0.84735040	Yes
FBXO5	0.342099875	0.86331385	Yes
AURKA	0.262047052	0.86242520	No
CLSPN	0.129781887	0.79633610	No
CDC25C	-0.323945820	0.04854368	No
